# Effect of Precipitation Temperature on the Properties of Cellulose Ultrafiltration Membranes Prepared via Immersion Precipitation with Ionic Liquid as Solvent

**DOI:** 10.3390/membranes8040087

**Published:** 2018-09-25

**Authors:** Daria Nevstrueva, Arto Pihlajamäki, Juha Nikkola, Mika Mänttäri

**Affiliations:** 1 LUT School of Engineering Science, Lappeenranta University of Technology, FI-53851 Lappeenranta, Finland; daria.nevstrueva@lut.fi (D.N.); mika.manttari@lut.fi (M.M.); 2 VTT Advanced Materials, P.O. Box 1300, FI-33101 Tampere, Finland; juha.nikkola@upm.com

**Keywords:** cellulose, ionic liquid, ultrafiltration, coagulation bath temperature, immersion precipitation

## Abstract

Supported cellulose ultrafiltration membranes are cast from a cellulose-ionic liquid solution by the immersion precipitation technique. The effects of coagulation bath temperature and polymer concentration in the casting solution on the membrane morphology, wettability, pure water flux, molecular weight cut-off, and fouling resistance are studied. Scanning electron microscopy, contact angle measurements, atomic force microscopy, and filtration experiments are carried out in order to characterise the obtained ultrafiltration cellulose membranes. The results show the effect of coagulation bath temperature and polymer concentration on the surface morphology and properties of cellulose ultrafiltration membranes. Optimisation of the two parameters leads to the creation of dense membranes with good pure water fluxes and proven fouling resistance towards humic acid water solutions.

## 1. Introduction

Polymeric membranes are widely utilised products today, with many applications in industry and everyday life. Currently, these devices can be stated to be a powerful tool for water purification, wastewater treatment, and drinking water production [1,2]. For these purposes, varieties of membranes have been created out of different polymers and inorganic materials with a few well-studied preparation techniques. One of the best-known and well-established membrane preparation processes is immersion precipitation. With this diffusion-induced technique, a wide range of different polymers (both fossil- and nature-based) can be transformed into the form of films or membranes [3,4].

In brief, the immersion precipitation process begins with the right choice of polymer and solvent to prepare a casting solution. Membrane creation is initiated when a finely distributed layer of casting solution is immersed into a coagulation bath filled with nonsolvent. To enable a phase inversion process, the solvent and nonsolvent should be miscible, so that the solvent in the distributed casting solution will be replaced by the nonsolvent. The replacement (diffusion) process results in the formation of polymeric membranes with a dense top layer and a porous sub-layer. The most important factors affecting the final structure of the membrane are the coagulation bath temperature and casting solution composition. The correct choice of these two factors allows controlling the solvent/nonsolvent exchange rate in the coagulation bath. It has been shown previously that systems with a rapid exchange rate between the solvent and nonsolvent lead to the formation of macrovoids in the membrane matrix [5,6,7]. On the other hand, systems with a slow diffusion rate have a tendency to form sponge-like membrane matrixes [8,9,10].

There are several fossil-based polymers used in membranes. They are often easy to use in film formation processes, but they also have some disadvantageous properties like hydrophobicity. Cellulose, considered the most abundant Earth biopolymer, would be good membrane polymer due to its hydrophilicity, renewability, biodegradability, and strong chemical structure. It already has a long history of utilisation in membrane technology [1,11]. On the other hand, this material is also well known for a difficult process ability into films, due to limited number of possible solvents. This is understood to originate from the great number of intra- and inter-chain hydrogen bonds. Unfortunately, almost all existing and utilised traditional organic solvents and solvent systems for cellulose dissolution and further shaping are toxic and difficult in solvent recovery, as well as produce waste [12]. Therefore, to get the full benefits of the unique properties of cellulose in membrane preparation, new solvents for cellulose should be introduced.

During the past ten years, several review articles about cellulose dissolution have been published [13,14], including recently [15,16,17]. The dissolution systems (non-derivatising) can be divided into three categories: aqueous, non-aqueous, and ionic liquids (ILs).

Ionic liquids (molten salts consisting of anions and cations) are non-flammable, recyclable, and chemically and thermally stable chemicals. Since the very first steps taken in the dissolution of cellulose in ILs [18], many types of ILs have been introduced as possible cellulose solvents [19,20,21,22,23,24]. The most common and investigated ones are 1-butyl-3-methylimidazolium chloride [bmim]Cl, 1-ethyl-3-methylimidazolium acetate [emim][OAc], and 1-allyl-3-methylimidazolium chloride [amim]Cl [25,26,27,28,29].

The introduction of new polymers and solvents for the formation of membranes has led to some lack of knowledge in the preparation process, and therefore it is important to investigate how the coagulation bath temperature (CBT) and casting solution composition affect the morphology and properties of the membrane. The preparation process of a cellulose membrane with IL was established in our previous study [30]. In the current study, the control of coagulation temperature and different concentrations of polymer in the casting solution are studied systematically. These factors have been investigated earlier for membranes prepared from solutions with organic solvents [5,31], but not for cellulose membranes precipitated from IL solutions. Cellulose is known to form crystals inside solid phase during the precipitation process. These crystals are not favourable for water passage through the cellulose film. Therefore, it would be beneficial to be able to control crystal formation during the precipitation process. In this work, cellulose ultrafiltration membranes are prepared via immersion precipitation with cellulose as polymer, ionic liquid ([emim][OAc]) as solvent, and deionised water as nonsolvent. The influence of coagulation bath temperature and casting solution concentration on the properties of the cellulose ultrafiltration membrane is investigated.

## 2. Materials and Methods

Cellulose with the degree of polymerisation 780 and α-cellulose content >93% was obtained from Domsjö pulp mill, Sweden, and used for membrane preparation. Ionic liquid 1-ethyl-3-methylimidazolium acetate ([emim][OAc]) was purchased from BASF, Ludwigshafen, Germany (Basionics^TM^ BC01, CAS: 143314-17-4, assay > 98%) and used as received for the preparation of the membrane casting solution. Non-woven material (PET, grade 3329, Ahlstrom Filtration LLC, Mt Holly Springs, PA, USA) was used as the support for all membrane samples. Humic acid (HA) and dextran (with different molar masses of 20, 40, 70, and 150 kg/mol) were supplied by Sigma-Aldrich (Steinheim, Germany) and Pharmacosmos (Holbaek, Denmark), respectively. HA (tech., cat.: H1, 675-2) water solutions with a concentration of 200 ppm were used as the model solution during the membrane rejection tests. Dextran water solutions with 200 ppm concentration were used to determine the molecular weight cut-off (MWCO) of the cellulose membranes. Deionised water obtained from an ELGA Labwater Centra-R120 water purification unit (ELGA Veolia, High Wycombe, UK) equipped with a deionisation cartridge (*ρ* > 15 MΩ·cm), was used for the preparation of all membrane samples (as a nonsolvent), membrane storing, and filtration tests.

The membranes were prepared by the phase inversion method: immersion precipitation [2]. This preparation process was investigated, established, and reported in our previous work [30]. The cellulose samples were ground before further use. Scissor-trimmed cellulose squares were downloaded into a Retsch PM100 planetary ball mill (Retsch GmbH, Haan, Germany) and ground for 20 min. Collected samples were dried at 45 °C for 24 h before actual use. The obtained polymer material was mixed with IL and allowed to dissolve for 12 h at 90 °C under vigorous stirring with a vertical kneader.

After a light yellow and transparent cellulose solution was obtained, it was placed under a vacuum for the degassing process. A thin layer of the degassed solution was poured onto the non-woven support material. It was distributed with an adjustable casting knife (BYK Additives & Instruments, Geretsried, Germany) at the controlled distribution pace of 50 mm/s by using an automatic film applicator machine (BYK Additives & Instruments, Geretsried, Germany). The nominal film casting thickness was chosen to be 250 µm. Right after the formation of the thin film layer, the obtained samples were immersed in a coagulation bath with deionised water at different temperatures (0, 10, 19, 35, 50, and 60 °C). The obtained membranes were first kept at the corresponding temperatures for 30 min. Then, all samples were transferred into water storage at about 22 °C for 24 h for complete removal of leftover ionic liquid from the cellulose films. Afterwards, the prepared supported cellulose membranes were stored in deionised water to prevent the samples from drying. All the created samples were given code names according to the casting solution concentration and coagulation bath temperature (CBT) used in sample creation. As an example, M7/0 was the code name for the membrane cast from a 7% cellulose solution in the ionic liquid at 0 °C CBT.

The cross-section and surface morphology of the prepared samples were examined with a JEOL JSM-5800 Scanning Electron Microscope (SEM, JEOL, Peabody, MA, USA) at an accelerating voltage of 10 kV. All membranes were taken out of the water storage and dried in room temperature conditions for 24 h before the actual SEM analysis. The room-dried samples were deposited in an oven at 45 °C for 2 h to remove any moisture in order to assure better conditions during the microscope process. The dry membrane samples were cut into an appropriate size and sputter-covered with a thin layer of gold.

The surface topographies were characterised by using non-contact mode atomic force microscopy (NC-AFM, Park Systems, Suwon, Korea). The NC-AFM analysis was performed by using Park Systems XE-100 AFM equipment (Park Systems, Suwon, South Korea) with cantilever 905M-ACTA (AppNano Inc., Mountain View, CA, USA). The typical scan rate was 0.4–0.6 Hz and the measured area was 1 µm × 1 µm. Three replicate measurements were performed to determine the roughness values and root mean square roughness value (RMS).

The membrane surface wettability (contact angle) was examined with an optical tensiometer (Theta model, Attension, Biolin Scientific AB, Gothenburg, Sweden) by using the sessile drop method at room temperature. All membrane samples were oven-dried (at 45 °C) and collected for contact angle determination. For hydrophilicity/hydrophobicity evaluation, a deionised water drop (3 µL) was automatically deposited by the system syringe on the membrane samples. The contact angle was measured at the surface-water-air interphase at room temperature within 10 s of the addition of the water drop. For each sample, five random locations were taken to minimise possible measurement error. The obtained contact angle results were calculated automatically and the mean value was used. The contact angle values were corrected for the surface roughness using the Wenzel approach [32].

Prior to the actual evaluation tests of membrane performance, all samples were stored in purified water to eliminate any residue of ionic liquid. The performance of the prepared membranes was characterised by using an in-house-made cross-flow setup described in our previous work [30]. This setup was equipped with two parallel filtration cells with an active membrane area of 12 cm^2^. Before the start of the actual pure water flux (PWF) measurements, precompaction filtration was carried out for every membrane sample. The samples were compacted with deionised water for 30 min at a pressure of 3 bar until stable flux values were achieved. All further filtration experiments were run at room temperature and a transmembrane pressure of 1 bar. The pure water fluxes (*F*) were calculated according to Equation (1):

(1) $$F=\frac{Q}{A\cdot \Delta t}$$

where *Q* is the quantity of collected permeate (kg), *A* is the active membrane area (m^2^), and Δ*t* is the sampling time (h).

The molecular weight cut-off (MWCO) determination tests were carried out on the same cross-flow setup. Dextran water solutions (200 ppm dextran in deionised water) with different molar masses were used to characterise the MWCO of the prepared membranes. Permeate and feed samples were collected for the determination of concentrations in a Shimadzu TOC-5050 (Shimadzu Co., Tokyo, Japan). The total organic carbon (TOC) analyser was also used for the determination of concentration of humic acid. The retention (*R*) results were calculated from Equation (2):

(2) $$R=\left(\frac{c_{f}-c_{p}}{c_{f}}\right)\cdot 100\%$$

where *c_f_* and *c_p_* are the concentrations of solute in the feed and in the permeate, respectively.

Filtration tests with humic acid (HA) (200 ppm) were carried out in the same cross-flow set as the other filtration tests. The feed tank was filled with a fouling solution (HA in deionised water). The system was left circulating for 24 h with 1 bar pressure. Pure water fluxes were measured before and after HA filtrations to characterise membrane anti-fouling abilities towards humic acid solutions.

## 3. Results

### 3.1. Membrane Morphology

**Figure 1:**
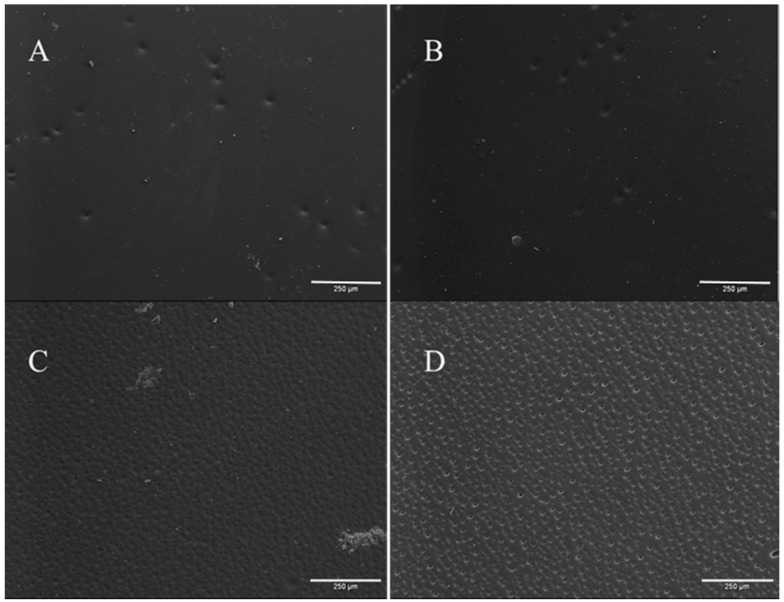
Scanning electron microscopy micrographs for 8% cellulose membrane (M8) surfaces precipitated at different coagulation bath temperatures (CBTs) ((**A**) 19 °C; (**B**) 35 °C; (**C**) 50 °C; (**D**) 60 °C). Scale bar 250 µm.

**Figure 2:**
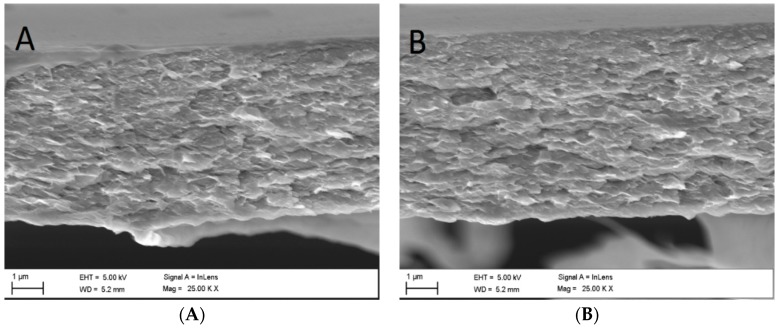
Scanning electron microscopy micrographs of 8% cellulose membrane cross-sections precipitated at (**A**) 19 °C and (**B**) 60 °C.

Scanning electron microscopy (SEM) micrographs of different sample surfaces (Figure 1) and cross-sections (Figure 2) were taken in order to observe the influence of different coagulation bath temperatures (CBTs) on the cellulose membrane morphology and to explain the filtration results obtained during membrane performance tests. It can be clearly seen in Figure 1 that, as the CBT values increased, membrane surface roughness and heterogeneity increased gradually. These results can be related to the filtration results presented in Section 3.2.

Cross-section morphologies of the cellulose membranes prepared at two different coagulation bath temperatures are shown in Figure 2. As can be seen in the SEM micrographs, the analysed samples appear to have nodular dense-layered structures. This may indicate that although CBT had a significant effect on membrane surface formation, it had less influence on the formation of the membranes’ inner layers. As Figure 2 shows, the increase in CBT from 19 to 60 °C results in the formation of membranes with a less dense nodular structure. However, the difference in density is minimal and can be only seen as a slightly thicker cross-section in the sample precipitated at 60 °C.

**Figure 3:**
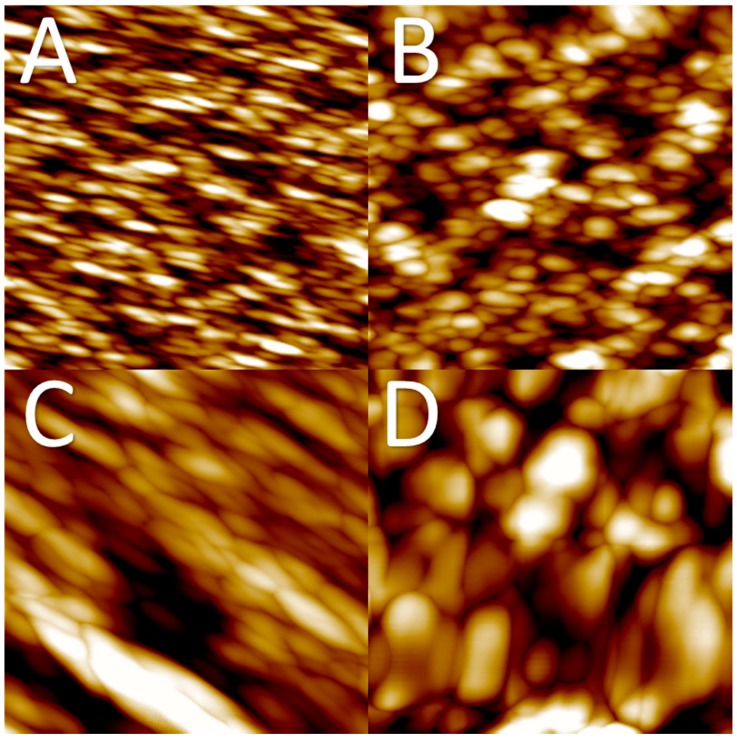
Atomic force microscopy (AFM) topography images for ultrafiltration membrane surfaces precipitated at different coagulation bath temperatures (CBTs) ((**A**) 19 °C, average RMS roughness 4 nm; (**B**) 35 °C, 7 nm; (**C**) 50 °C, 10 nm; (**D**) 60 °C, 30 nm). Scan size 1 µm × 1 µm, Z-range 30 nm.

Figure 3 presents atomic force microscopy (AFM) topography images for ultrafiltration membrane surfaces precipitated at different CBTs ((A) 19 °C; (B) 35 °C; (C) 50 °C; (D) 60 °C). The AFM images show differences between the membrane surfaces. White spots in the figures represent higher peaks (hills), whereas darker spots represent lower places (valleys). In Figure 3A, these areas are small and distributed evenly, indicating a smoother surface. On the other hand, in Figure 3C,D these areas (both white and dark) have become larger, showing an increase in surface roughness. It was found that the lowest precipitation temperature resulted in the lowest surface roughness. Moreover, the increase in precipitation temperature led to a rougher surface.

**Figure 4:**
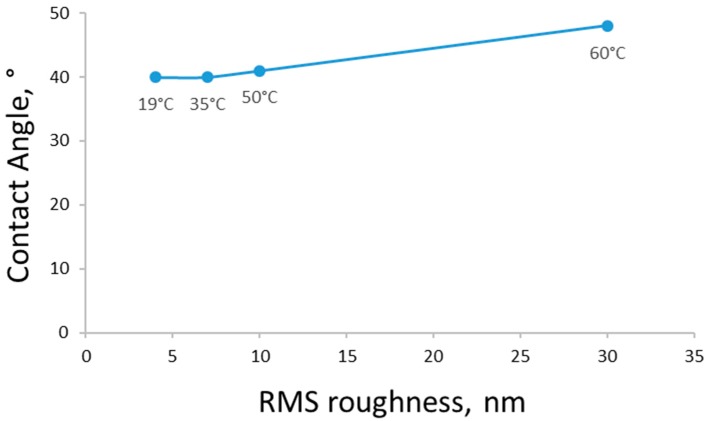
Roughness corrected contact angle and RMS roughness data for 8% cellulose membranes precipitated at different coagulation bath temperatures (CBTs).

The relation between the membrane contact angles and surface roughness is shown in Figure 4. It was not expected that the membrane hydrophilicity would change a lot. However, with increasing CBT, the root mean square (RMS) value increased, which led to measured contact angle showing a descending trend. However, roughness corrected contact angles indicate increase in hydrophobicity when precipitated at higher CBTs.

### 3.2. Filtration Studies of Cellulose Membranes

**Figure 5:**
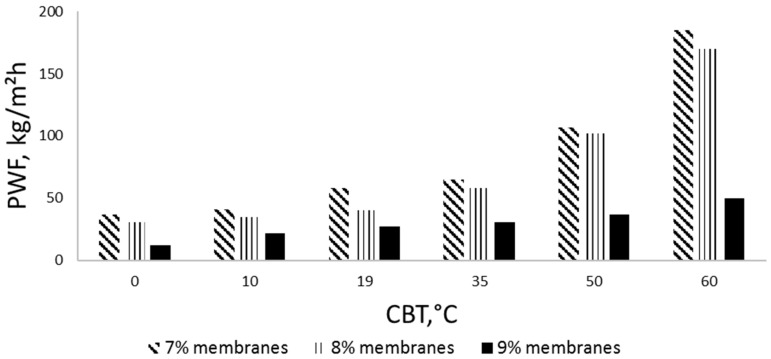
Pure water fluxes (PWFs) for 7–9% cellulose membranes, precipitated at different coagulation bath temperatures (CBTs). Filtration pressure 1 bar.

**Figure 6:**
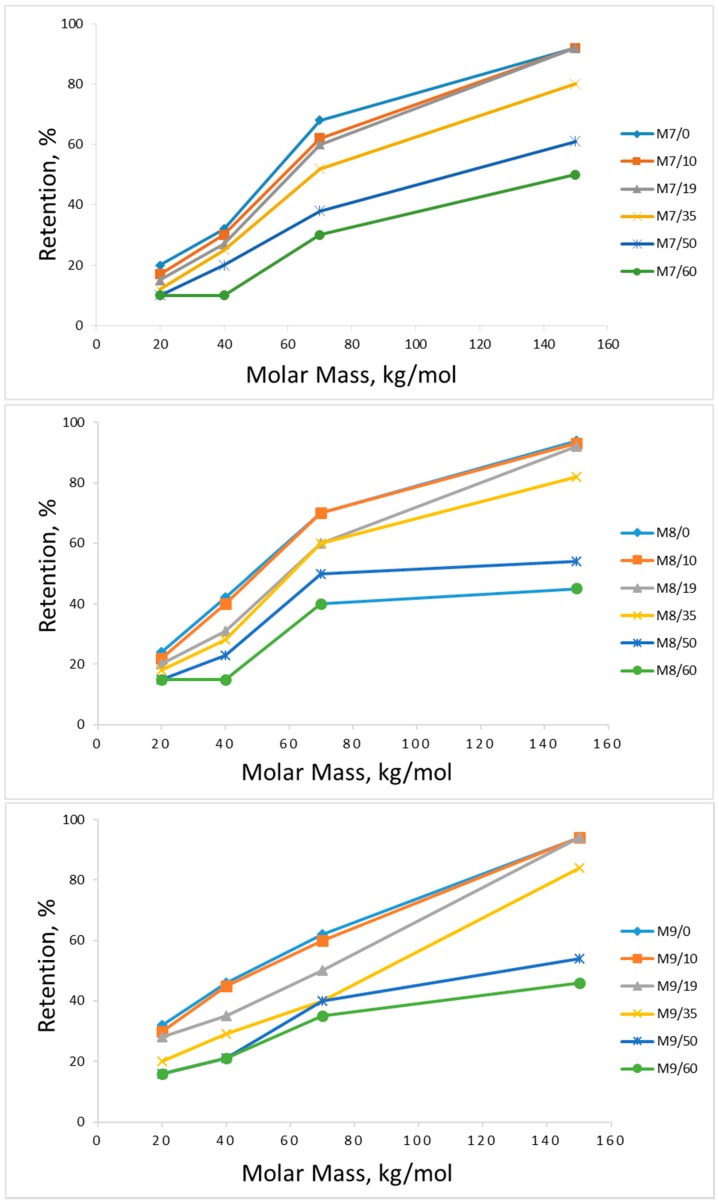
Dextran retentions of prepared membranes as a function of model solution dextran molar mass. The membranes were made from 7–9% cellulose solutions at indicated coagulation bath temperatures (CBTs) (M9/60 = membrane casted from 9% cellulose solution at 60 °C, etc.). Filtration pressure 1 bar.

**Figure 7:**
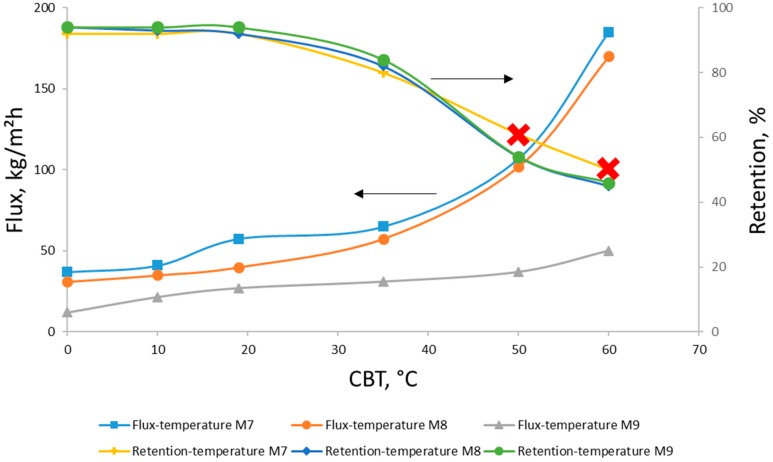
Flux and retention results for aqueous solutions of dextran 150 kg/mol measured for 7–9% cellulose membranes (M7-M8-M9) precipitated at different coagulation bath temperatures (CBTs). Important notice: although retention data for membranes M7/50 and M7/60 is included in the graph, the samples were mechanically unstable during the filtration experiments.

The effect of the CBT on the pure water flux (PWF), the fluxes of the dextran model solution, and the retention of various molar mass dextrans is presented in Figure 5, Figure 6 and Figure 7. As can be seen, the increase in the coagulation bath temperature leads to growth in the pure water fluxes and a decrease in the model compound retention. This tendency is proven by the values obtained for the membrane samples with different concentrations, and can be explained with consideration of previous work done in this field [5,30], and the membrane morphology observations made in this work via SEM (Figure 1 and Figure 2).

It should be mentioned that the dextran compounds used in the filtration experiments were of a technical grade, with up to 10% of lower molar mass impurities. While the main separation mechanism for ultrafiltration membranes is sieving [33], it is quite expectable for the retention of technical grade dextran to never get to 100% because of the lower molar mass molecules passing through the membrane.

**Figure 8:**
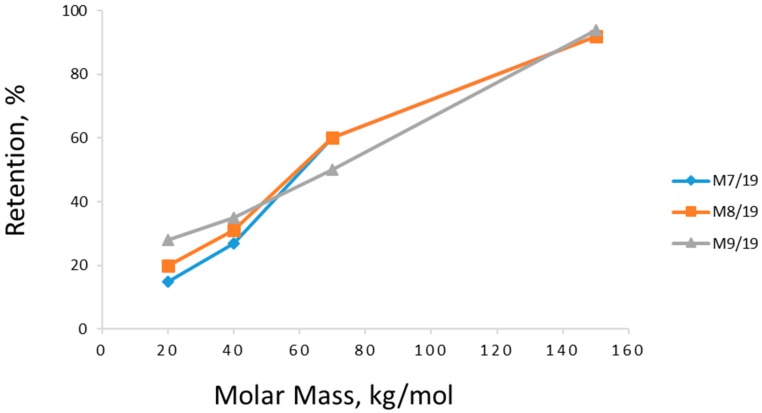
Effect of cellulose concentration in the membrane casting solution on the retention of dextran molecules. M7/19 stands for 7% cellulose in casting solution and coagulation temperature 19 °C. All filtration experiments were done at room temperature with filtration pressure at 1 bar.

The effect of polymer concentration in the casting solution on the membrane performance was also observed during the filtration experiments. As can be seen in Figure 7 and Figure 8, the membranes cast from the 8% cellulose solution (M8) with mild CBT conditions (0, 10, 19 °C) show the best PWF without any loss in model compound retention. The samples precipitated in higher temperatures show an increase in PWF with a loss in the retention ability and mechanical stability (part of the membranes degraded during the filtration experiments). The membrane performance starts to have significant changes when CBT is 35 °C or more, especially in model compound retention. PWFs start to increase too at the same CBT. The 9% membranes (M9) have a much lower PWF compared to the other samples. It should also be mentioned that a higher concentration of polymer in the casting solution may lead to difficulties in polymer dissolution and membrane casting. Cellulose–ionic liquid solutions are reported to be very viscose [18,20], which may cause problems during solution distribution on the support material. In addition, as mentioned above, all 9% membranes appeared to be very dense-structured according to the SEMs. By contrast, the 7% cellulose membranes (M7) showed the highest PWF with the lowest retention ability towards the model compound solution at lower CBTs, and high but very unstable retention for 50 °C and 60 °C. The samples with 7% of cellulose in the casting solution, cast and precipitated at higher temperatures, degraded during the filtration processes if the filtration time was more than 1 h. This pattern can be explained with the lack of cellulose in the casting solution, which led to the formation of fragile, defective membranes with polymer-poor areas during the precipitation process.

### 3.3. Membrane Anti-Fouling Properties

Membrane fouling is one of the most essential problems in the utilisation of membrane technology on an industrial scale. Membranes with a low fouling tendency are highly required in industrial purification processes. The new cellulose membranes obtained in this study were tested with a humic acid (HA) water solution in a concentration of 200 ppm to determine their possible anti-fouling properties towards the model compound. The choice of the fouling agent can be explained by the wide utilisation of humic acid compounds in membrane characterisation [33]. All filtration tests were carried out for 8% membranes (M8) prepared at a lower coagulation bath temperature (CBT), because these samples have shown the best pure water flux (PWF) and retention results for model compounds in earlier evaluations. The PWFs were measured before and after 24 h of HA solution filtration and were found to be identical. It should also be mentioned that the retention of HA for all the tested samples was 70–80%.

## 4. Discussion

To explain the observed contrast in CBT influence on the outer and inner cellulose membrane layers’ formation, previous research conducted in the area of fossil-based polymers/organic solvents systems should be taken into consideration. According to the literature, the membrane morphology is highly dependent on not only the CBT effect, but also the polymer type, solvent, nonsolvent, and various additive choices for the membrane precipitation process [34]. Phase separation (immersion precipitation) has been proved to be a diffusion process based on thermodynamic instability in a polymer-solvent-nonsolvent system caused by mutual diffusive processes between all components. These diffusion processes are triggered by high miscibility between the solvent-nonsolvent and lack of miscibility between the nonsolvent-polymer. In the case of this study, the solvent (ionic liquid) was diffused into a coagulation bath filled with deionised water. On the other hand, nonsolvent (deionised water) diffuses into the cellulose polymer matrix. The exchange process between components lasts until the system reaches thermodynamic instability, which starts the liquid-liquid demixing process, leading to actual cellulose membrane formation [35,36].

The mechanism for porous film via phase separation in polymer-solvent-water systems has been identified to go in two possible ways: spinodal decomposition or nucleation. Despite having different starting phases, both mechanisms result in the same spherical (or nodular) membrane morphology. According to the SEM observations of cellulose membrane morphology (Figure 2), the membranes obtained in this study were formed via a spinodal decomposition process. Detailed studies of the behaviour patterns of cellulose while being precipitated in aqueous media from ionic liquid solutions have been previously reported on and investigated in earlier works of various research groups [37,38,39].

In theory, the CBT should affect the diffusion velocity for spinodal decomposition and exchange between the phases drastically, causing a change in the membrane morphology. A high CBT should increase the diffusion rate between all components, forcing thermodynamic instability into the system. According to various studies [4,35], this should be the reason for the formation of membranes with macrovoids. However, in the SEM cross-section micrographs, only membranes with a dense structure can be seen. This may once again be explained by two possible assumptions. First, the CBT is not the only parameter affecting the membrane structure during phase separation. The type of solvent, the polymer concentration, and viscosity of the casting solution can also be considered as factors that affect the membrane morphology. The suppression of macrovoid formation in this work and the development of dense cellulose membrane structures were due to the high viscosity of the casting solution (the viscosity can be hundreds of times higher than generally in organic solvents) for all the tested polymer concentrations. In this case, the diffusion processes involving the solvent and nonsolvent took a longer time even at a higher CBT. A slower diffusion hinders the demixing rate and causes delayed solidification of the membrane film. Hence, the high casting solution viscosity may have been the reason for the dense membrane formation (Figure 2).

However, it may be stated that the CBT in this study had a direct effect on the formation of the cellulose membrane surface. Although the influence of CBT on the membrane cross-sections was not clearly detected, the SEM micrographs of all sample surfaces show quite a clear pattern (Figure 1). With the increase of CBT, the surface macroscopic roughness increased significantly. These findings are in agreement with the results of membrane precipitation from common organic solvents [39]. With this, a second assumption can be made: that the immediate formation of surface layers would have had a direct suppressive effect on membrane inner layer formation. A solidified cellulose surface may prevent ionic liquid-water exchange during immersion precipitation, leading to diffusion rate suppression.

AFM topography revealed a typical granulated structure of the regenerated cellulose film. These observations are congruent with previous findings in the area of regenerated cellulose films [36]. Apparently, the crystal size tends to increase with the increase in temperature. This would present an opportunity to control cellulose crystallisation during the membrane formation process. The findings from the AFM topography images are in agreement with the SEM images for membrane surfaces and contact angle measurements (Figure 4).

It is well known that PWF values depend on the membrane structure (thickness, mean pore size and porosity) [1]. The type and structure of the membrane depend on the precipitation parameters: the coagulation bath temperature, polymer concentration in the casting solution, and the solvent-nonsolvent pair. The cellulose membranes precipitated in this study with low CBTs (0, 10, 19 °C) showed a relatively low PWF and high rejection ratio for the dextran model solutions. All the samples prepared at higher CBTs (35, 50 and 60 °C) had much higher PWF results with almost no retention even for the highest molar mass dextran. All the aforementioned can be taken as evidence of the direct influence of the CBT on the membrane filtration properties. The decrease in model compound retention of all membranes (7%, 8% and 9%) at higher CBTs (50 and 60 °C) is likely the result of a more open polymer matrix structure formed during rapid precipitation process. Too rapid precipitation could cause larger pores and more porous cellulose films.

On the other hand, precipitation at the higher CBT values led to a reduction of membrane durability during the filtration experiments. All the samples precipitated at 50 and 60 °C were very fragile, with a tendency to break in the cross-flow filtration set-up. The mechanical instability of these samples can be explained with fast and vulnerable precipitation affected by the rapid demixing process in the coagulation bath.

In addition to CBT and polymer concentration, the hydrophilicity of the membrane surface also affects the membrane filtration performance. The main factor affecting the retention ability of an ultrafiltration membrane is the membrane morphology, due to the sieving mechanism of this filtration type [1]. However, the general factor defining the permeability of a cellulose membrane may not be only the morphology, but also the hydrophilicity of the chosen material. Cellulose is a naturally hydrophilic polymer with initially low contact angles, which was proven by the contact angle measurements carried out in this study. The results for the roughness corrected contact angles of the membranes precipitated from the 8% cellulose solution in ionic liquid can be found in Figure 5. It should be stated that different CBTs had minimal effects on the membrane contact angles at lower temperatures. Overall, all the tested samples were significantly hydrophilic. However, for the samples prepared at lower CBTs and higher polymer concentration, the PWFs were not so high, and the retention for dextran solutions was also low. So, in this case, the membrane morphology (all the samples were dense) played a significant role. After all the filtration experiments, it can be concluded that membrane filtration performance and retention abilities are a combined result of the effects of the CBT, polymer concentration, and solvent choice.

The HA solution filtration experiments showed no fouling of the cellulose membranes. They also confirmed the dextran retention test results, where the higher CBT values led to a lower rate of model compound retention. Therefore, a decrease in the HA solution rejections with growth in the CBT was expected (80% retention for membranes formed at CBT of 0 °C and 70% retention at CBTs of 50 and 60 °C). The low fouling tendency of cellulose membranes can be explained by the high natural hydrophilicity of cellulose.

In almost all analyses and tests we could observe significant changes in the membrane properties at the coagulation temperature of about 35–40 °C (see Figure 4, Figure 5, Figure 6, Figure 7 and Figure 8). This was most likely related to the glass transition temperature (T_g_) of cellulose. The T_g_ of dry cellulose has been determined by differential scanning calorimetry (DSC) to be about 220 °C [40]. However, just a small amount of water plasticises the cellulose and the T_g_ decreases rapidly (the T_g_ of cellulose with 5% water content is around 80 °C [40]). When a membrane is formed in the phase transition process, pores are formed by the merging of free volume between polymer chains. This pore formation involves some movements of the polymer chains or segments. In this movement of segments, a clear change at T_g_ can be seen, resulting in much faster movements at higher temperatures than T_g_. Also, the free volume expands above the T_g_. This would lead to larger pores in the membranes created at temperatures higher than the polymer T_g_ [41]. The cellulose membranes in this study were cast out of a hot solution and coagulated in water baths at temperatures of 0 to 60 °C. Therefore, the T_g_ of the cellulose polymer was probably somewhat higher than 40 °C, which fits well for the cellulose with water content of about 5–6% [40].

## 5. Conclusions

The purpose of this study was to evaluate the effect of temperature during the precipitation process and polymer concentration in the casting solution on the morphology and properties of cellulose ultrafiltration membranes. Various membranes with different concentrations of cellulose in [emim][OAc] ionic liquid and coagulation bath temperatures were prepared. The obtained samples were characterised by their pure water flux, model compound retention, morphology, water contact angle, and anti-fouling properties. According to the test evaluations, an increase in the coagulation bath temperature always resulted in an increase of pure water flux and a decrease in model compound retention. In general, the effect of coagulation bath temperature had a clear predominant impact on the morphology (membrane surface) and filtration properties of all samples in comparison to the casting solution concentration. The obtained cellulose membranes exhibited perfect humic acid-fouling resistance due to their hydrophilicity. In the findings of this work, the best membrane samples were prepared by using 8% cellulose concentration at a temperature of 19 °C. These samples showed the best combination of flux and retention of the model compound and humic acid solutions.
